# Microbial Biodegradation of Paraffin Wax in Malaysian Crude Oil Mediated by Degradative Enzymes

**DOI:** 10.3389/fmicb.2020.565608

**Published:** 2020-09-08

**Authors:** Nur Aina Adlan, Suriana Sabri, Malihe Masomian, Mohd Shukuri Mohamad Ali, Raja Noor Zaliha Raja Abd Rahman

**Affiliations:** ^1^Enzyme and Microbial Technology Research Centre, Faculty of Biotechnology and Biomolecular Sciences, Universiti Putra Malaysia, Serdang, Malaysia; ^2^Department of Microbiology, Faculty of Biotechnology and Biomolecular Sciences, Universiti Putra Malaysia, Serdang, Malaysia; ^3^Centre for Virus and Vaccine Research, School of Science and Technology, Sunway University, Bandar Sunway, Malaysia; ^4^Department of Biochemistry, Faculty of Biotechnology and Biomolecular Sciences, Universiti Putra Malaysia, Serdang, Malaysia

**Keywords:** crude oil, paraffin wax, biodegradation, thermophilic bacteria, alkane monooxygenase, alcohol dehydrogenase, lipase, esterase

## Abstract

The deposition of paraffin wax in crude oil is a problem faced by the oil and gas industry during extraction, transportation, and refining of crude oil. Most of the commercialized chemical additives to prevent wax are expensive and toxic. As an environmentally friendly alternative, this study aims to find a novel thermophilic bacterial strain capable of degrading paraffin wax in crude oil to control wax deposition. To achieve this, the biodegradation of crude oil paraffin wax by 11 bacteria isolated from seawater and oil-contaminated soil samples was investigated at 70°C. The bacteria were identified as *Geobacillus kaustophilus* N3A7, NFA23, DFY1, *Geobacillus jurassicus* MK7, *Geobacillus thermocatenulatus* T7, *Parageobacillus caldoxylosilyticus* DFY3 and AZ72, *Anoxybacillus geothermalis* D9, *Geobacillus stearothermophilus* SA36, AD11, and AD24. The GCMS analysis showed that strains N3A7, MK7, DFY1, AD11, and AD24 achieved more than 70% biodegradation efficiency of crude oil in a short period (3 days). Notably, most of the strains could completely degrade C_37_–C_40_ and increase the ratio of C_14_–C_18_, especially during the initial 2 days incubation. In addition, the degradation of crude oil also resulted in changes in the pH of the medium. The degradation of crude oil is associated with the production of degradative enzymes such as alkane monooxygenase, alcohol dehydrogenase, lipase, and esterase. Among the 11 strains, the highest activities of alkane monooxygenase were recorded in strain AD24. A comparatively higher overall alcohol dehydrogenase, lipase, and esterase activities were observed in strains N3A7, MK7, DFY1, AD11, and AD24. Thus, there is a potential to use these strains in oil reservoirs, crude oil processing, and recovery to control wax deposition. Their ability to withstand high temperature and produce degradative enzymes for long-chain hydrocarbon degradation led to an increase in the short-chain hydrocarbon ratio, and subsequently, improving the quality of the oil.

## Introduction

Crude oil is an important commodity and a central source of energy in the world. It is predominantly composed of 50 to 80% petroleum hydrocarbons, i.e., alkanes, cycloalkanes, and aromatic alkanes content (Yan et al., 2013). Based on the hardness, crude oil is classified into high, moderate, and low wax content crude oil (Doerffer, 1992). The high wax content in waxy crude oil is due to the presence of a large amount of paraffin wax. Paraffins with carbon chain length higher than C_18_H_38_ are regarded as waxes and could solidify during the production and transportation stages of the crude oil (Bai and Zhang, 2013). The crystallization of waxes happens due to the change in temperature and pressure in the pipeline. The continuous wax build-up obstructs oil flow and leads to marginal oil recovery and the shutdown of the production pipeline (Sharma et al., 2016). On top of that, oil companies lose millions of dollars annually due to the reduced oil production and high cost of wax removal (Hosseinipour et al., 2014). The flow assurance problems also reflect the rising cost of production because extra horsepower and workforce are needed to alleviate the pressure to prevent wax deposition (Elsharkawy et al., 2000).

Current methods to mitigate paraffin wax deposition are by thermal (e.g., insulation and hot oil treatment), mechanical (e.g., scrapers and pigs) and chemical treatments (e.g., solvents, wax inhibitors, and dispersants) or a combination of these treatments (Jennings and Breitigam, 2010; Towler et al., 2011). However, there are certain disadvantages of using these remedies such as expensive cost for long production tubes (Sakthipriya et al., 2017), toxicity issues (Sadeghazad and Ghaemi, 2003; Al-Yaari, 2011), long-term damage to the pipelines and wells (Al-Yaari, 2011), and production downtime (Sood and Lal, 2008). Due to the abovementioned problems, an alternative approach to prevent wax deposition, such as green technology using microbes, is needed.

Microbes play essential roles in bioremediation of oil, and many studies have been published on microbial remediation to degrade paraffin wax in crude oil. However, most of these microorganisms were mesophilic bacteria such as *Bacillus cereus* QAU68 and *Bacillus subtilis* XCCX (Liu et al., 2013); *Pseudomonas aeruginosa* B1 and *Acinetobacter junii* B2 (Liu et al., 2012); and *Rhodococcus* sp. Moj-3449 (Binazadeh et al., 2009). Nevertheless, the operating temperature in the pipeline and petroleum reservoirs sometimes exceeds 50°C. Therefore, thermophilic hydrocarbon degraders are of particular significance. Thermophilic bacteria could survive the high temperature while degrading the crude oil. Some of the thermophilic bacteria capable of degrading crude oil that has been reported are *Geobacillus kaustophilus* TERI NSM (Sood and Lal, 2008), *Geobacillus pallidus* H9 (Wenjie et al., 2012), and *Geobacillus* sp. ZY-10 (Sun et al., 2015). Although thermophilic *Geobacillus* spp. had been studied previously, however, some of them were grown at longer incubation period such as 8, 10, or 22 days (Wang et al., 2006; Zhang et al., 2012; Fan et al., 2020). Furthermore, the majority of the research on *Geobacillus* spp. oil degradation reported a shorter range of hydrocarbon decomposition. As an example, *Geobacillus* sp. SH-1 was able to degrade *n-*alkanes from C_12_ to C_33_ (Zhang et al., 2012), *Geobacillus toebii* B-1024 and *Geobacillus* sp. 1017 were able to degrade C_10_–C_30_ and C_13_–C_19_, respectively (Tourova et al., 2016), while *Geobacillus stearothermophilus* A-2 could partially degrade long-chain alkanes (C_22_–C_33_) (Zhou et al., 2018). Therefore, thermophilic bacteria that could degrade more long-chain hydrocarbon in a shorter period while increasing the short-chain hydrocarbon is an advantage.

Biodegradative enzymes present a significant role in microbial degradation of petroleum hydrocarbons (Yong and Zhong, 2010; Wasoh et al., 2019). The hydrocarbon removal efficiency depends on the bacteria used and their enzymatic oxidation characteristics (Elumalai et al., 2017). In the alkane degradation pathway, the degradation of *n*-alkane is usually initiated by alkane hydroxylase enzymes that transform alkane into alkanols by targeting different ranges of hydrocarbon (van Beilen et al., 1994; Singh et al., 2012). The terminal, biterminal, and subterminal oxidation pathways have been known and verified in bacteria from different genera (Ji et al., 2013). In the terminal oxidation pathway, the alkanes yielded corresponding alcohols after the initial attack at the terminal methyl group. Alcohol dehydrogenases and aldehyde dehydrogenases further oxidize the substrates to fatty acids, which then enters ß-oxidation (Watkinson and Morgan, 1990). In biterminal oxidation, the cleaved termini of *n*-alkane would undergo oxidation, forming the corresponding fatty acid without rupturing the carbon chain (Ji et al., 2013). In this pathway, ω-hydroxy fatty acid produced during terminal oxidation is converted to a dicarboxylic acid, which also enters ß-oxidation (Coon, 2005). Primary and secondary alcohol or methyl acetone are produced in subterminal oxidation. Ketone produced from the conversion of secondary alcohol is oxidized by Baeyer-Villiger monooxygenase to form an ester. Hydroxylation by an esterase would then generate alcohol and fatty acid (Kotani et al., 2007).

Lipases and esterases are also important enzymes in monitoring the biodegradation of crude oil (Kadri et al., 2018b). Both of these enzymes can cleave the carboxylic ester bonds in fats and oils to release fatty acid and glycerol (Lee et al., 2015). The fatty acid can also function as a carbon source for bacteria, thus increasing the hydrocarbon degradation. Lipases and esterases differ in their substrate specificity. The long-chain esters are hydrolyzed by lipases, while the short-chain esters are hydrolyzed at various rates by esterases and lipases (Chahinian and Sarda, 2009). As crude oil degradation is a continuous process, the intermediate products from the conversion of *n*-alkanes along the pathway would produce the substrate needed for lipase and esterase enzymes to utilize. Hence, this would enable the monitoring of oil biodegradation.

This study focused on finding a novel thermophilic bacterial strain capable of degrading paraffin wax in crude oil at 70°C. The functions of the strains were compared in terms of crude oil degradation efficiency and enzyme activities. Efficient crude oil degradation by the bacterial strains could be an alternative solution to control wax deposition and further improve oil flow in the pipeline.

## Materials and Methods

### Crude Oil Samples

Crude oil with varying hardness was obtained from Petronas Research Sdn. Bhd., Malaysia. The characteristics of the crude oils were observed as follows; very hard, dense, and solid with 63°C pour point, and 66% long-chain alkanes composition (>C_20_) (oils A and B); hard, solid, and brittle with 48°C pour point, and 64% long-chain alkanes composition (>C_20_) (oils C and D); soft, watery, and semi-solid with 42°C pour point and 55% long-chain alkanes composition (>C_20_) (oil E). The higher composition of > C_20_ signifies the crude is waxier and have higher viscosity. The density of the crude oil is relatively similar (0.8 g/cm^3^).

### Microorganisms

Strains N3A7 and D9 were previously isolated from seawater sample at Kampung Cherating Lama, 26080 Balok, Pahang, Malaysia (4°7′15.80′′ N, 103°23′20.20′′ E) on October 2016. Strains MK7, DFY1, T7, and DFY3 were previously isolated from oil-contaminated soil samples at a car workshop in Kampung Sungai Tua Tambahan, Tanah Gantian, 68100 Batu Caves, Selangor, Malaysia (3°15′28.09′′ N, 101°40′38.65′′ E) on November 2016. Strains AZ72, SA36, NFA23, AD11, and AD24, were isolated at Passenger Jetty, Jalan Tanjung Kemuning, Kampung Bahasa Kapor, 71000, Port Dickson, Negeri Sembilan, Malaysia (2.5223° N, 101.7931° E) on September 2017. All strains were stock cultured at the Enzyme and Microbial Technology Centre (EMTech) Lab, Faculty of Biotechnology and Biomolecular Sciences, Universiti Putra Malaysia, Malaysia.

### Isolation and Screening of Hydrocarbon-Degrading Strains

An aliquot of 10 ml of the sample was pipetted into 250 ml Schott bottle containing 50 ml Bushnell and Haas (BH) broth medium supplemented with 2% (w/v) crude oil A, B, C, D, and E. The BH medium composed of (g/l): KH_2_PO_4_ (1 g), K_2_HPO_4_ (1 g), NH_4_NO_3_ (1 g), MgSO_4_ ⋅ 7H_2_O (0.2 g), FeCl_3_ (0.05 g), CaCl_2_ ⋅ 2H_2_O (0.02 g) and yeast extract (5 g). The cultures were incubated at 70°C at 200 rpm shaking rate for 7 days. A 100 μl aliquot of each bacterial culture was spread onto nutrient agar medium (Merck, Darmstadt, Germany) and incubated at 70°C for 18 h. Colonies with different morphologies were transferred onto fresh Nutrient Agar medium for three cycles and subsequently were preserved in Nutrient Broth (Merck, Darmstadt, Germany) using 20% (v/v) glycerol at −80°C.

The screening of paraffin wax degrading bacteria was made according to the method described by Patowary et al. (2016) with slight modifications. The bacteria were grown overnight on nutrient agar at 70°C. Single colonies were transferred into the nutrient broth, and the cultures were incubated overnight. The bacterial cultures were adjusted to OD_600_∼0.5. The bacterial inoculum (5%) were transferred into 250 ml Schott bottle containing 50 ml ocean water medium (OWM) (Kester et al., 1967) containing (g/l): yeast extract (5), peptone (5), beef extract (3), NaCl (19.9), KCl (0.6), MgCl_2_ ⋅ 6H_2_O (4.4), MgSO_4_ ⋅ 7H_2_O (5.8), CaCl_2_ ⋅ 2H_2_O (0.8), supplemented with 2% (w/v) crude oil B. The cultures were incubated at 70°C and 200 rpm shaking rate for 7 days. Uninoculated OWM supplemented with 2% (w/v) crude oil B served as abiotic control. The colony forming unit (CFU) value was monitored to determine the viable cell growth of the cultures.

### Bacterial Identification

The genomic DNA of the bacteria was extracted using DNeasy Blood and Tissue Kit (Qiagen, Hilden, Germany) according to the manufacturer’s instructions. The 16S rRNA genes were amplified using a set of universal primers, 8F (5′-AGAGTTTGATCCTGGCTCAG-3′) and 1492R (5′-ACGGCTACCTTGTTACGACTT-3′) (Weisburg et al., 1991). Each 50 μl of PCR reactions contained 25 μl of 2 × EasyTaq PCR Supermix (TransGen Biotech, China), 10 μM of each primer and 5 μl of genomic DNA as a template. Thermal cycling was performed in a G-storm GS1 thermal cycler (GRI, Ltd., Essex, United Kingdom) with the following parameters: initial denaturation step of 95°C for 1 min, followed by 30 cycles of 95°C for 15 s, 57°C for 15 s, and 72°C for 10 s. A final extension step consisting of 72°C for 5 min was included. Amplification products were checked by 1% (w/v) agarose gel electrophoresis. PCR amplicons were then sequenced (Apical Scientific Sdn. Bhd., Seri Kembangan, Malaysia). The sequences were analyzed and edited with Chromas Lite software (version 2.6.5; Technelysium Pty. Ltd., South Brisbane, QLD, Australia) and compared against the sequences in the National Center of Biotechnology Information (NCBI) database by using the BLASTn program^1^.

### Phylogenetic Analysis

Clustal Omega (Sievers and Higgins, 2017) program was used to create multiple alignments of nucleotide gene sequences, while MEGA 7.0 (Kumar et al., 2016) program was used to construct the phylogenetic tree. The phylogenetic tree was constructed using the neighbor-joining method (Saitou and Nei, 1987) with *p*-distance method (Nei and Kumar, 2000). The robustness of individual branches was estimated by bootstrapping with 1000 replications (Felsenstein, 1985).

### Growth Medium Optimization

An aliquot of 5% inoculum was added into a 250 ml Schott bottle containing 50 ml medium; M1 (OWM without organic nitrogen source), M2 (OWM with yeast extract only), M3 (OWM with peptone only), and M4 (OWM with beef extract only) medium. The M1–M4 media were supplemented with 2% (w/v) crude oil B. The cultures were subsequently incubated for 7 days at 70°C and 200 rpm shaking rate. Bacterial growth and crude oil degradation in the respective media were compared.

### GCMS Evaluation of Oil Biodegradation

The cultures were grown in 500 ml conical flask containing 50 ml M2 medium containing 2% (w/v) of crude oil B at 70°C and 150 rpm shaking rate. The flasks were secured with silicone seals on the cap to prevent the evaporation of some oil fractions. The non-inoculated medium was incubated under the same condition served as a control. After 3 days of incubation, the culture was mixed with *n*-hexane (1:1). The mixtures were vortexed for 5 min and transferred into a separatory funnel. The extract was allowed to separate for 10 min, and the upper layer was collected. A 100 μl aliquot of the extract was added to a vial containing 900 μl *n*-hexane and kept at −20°C until further use (Kaida, 2012).

The residual crude oil components were analyzed by GCMS Agilent 7890A using HP-5MS column (30 m × 0.25 mm ID × 0.25 μm film thickness) (Ameen et al., 2016). The GC program was set with a split ratio of 10:1. Helium was used as the carrier gas with a flow rate of 1 ml/min. The oven temperature was set at 35°C for 1 min and was increased 15°C/min to 160°C for 1 min and then 5°C/min to 315°C for 12 min. A total of 1 μl of the sample was injected. The MS source and quad temperature were set at 230°C and 150°C, respectively. The biodegradation efficiency (BE) percentage was determined according to Michaud et al. (2004):

$$\text{BE}\%=100-\left(\frac{\mathrm{The}\,\mathrm{total}\,\mathrm{peak}\,\mathrm{area}\,\mathrm{of}\,\mathrm{the}\,\mathrm{sample}}{\mathrm{The}\,\mathrm{total}\,\mathrm{peak}\,\mathrm{area}\,\mathrm{of}\,\mathrm{control}}\right)\,\times \,100$$

### Enzyme Assays

The bacterial extracellular enzymes were collected to determine the enzyme activities. The cultures were grown as described earlier. The supernatant of the cultures was collected every 24 h by centrifugation at 4°C and 8000 × *g* for 10 min.

#### Alkane Monooxygenase

Alkane monooxygenase activity was measured based on Jauhari et al. (2014) with slight modifications. The reaction mixture of 250 μl containing 145 μl of 100 mM PBS buffer (pH 7.0), 50 μl crude enzyme, 5 μl of 50 mM *n*-hexadecane and 50 μl of 125 μM of NADH was incubated at 70°C for 10 min with shaking. The reaction mixtures were then measured spectrophotometrically at 340 nm. One unit of alkane monooxygenase activity corresponded to the rate at which 1 μmol NADH is oxidized per min.

#### Alcohol Dehydrogenase

Alcohol dehydrogenase activity was measured based on Jauhari et al. (2014) with slight modifications. The reaction mixture of 250 μl containing 140 μl of 100 mM PBS buffer (pH 7.0), 50 μl crude enzyme, 10 μl of 25 mM of 1-hexadecanol and 50 μl of 1.65 mM of NAD^+^ was incubated at 70°C for 10 min with shaking. The reaction mixtures were then measured spectrophotometrically at 340 nm. One unit of alcohol dehydrogenase corresponded to the rate at which 1 μmol NADH is formed per min.

#### Lipase

Lipase activity was measured based on Zuo et al. (2010) with slight modifications. The reaction mixture contained 220 μl of 100 mM PBS buffer (pH 7.0), 25 μl of 5 mM of 4-nitrophenyl palmitate and 5 μl of the crude enzyme was incubated at 70°C for 10 min with shaking. The reaction mixture was added with Triton X-100 to prevent turbidity (Gupta et al., 2002). Immediate reading of the mixture using a spectrophotometer at 410 nm was performed. One unit of lipase activity was defined as the rate at which 1 μmol of *p*-nitrophenol is liberated per min under assay conditions (Rúa et al., 1997).

#### Esterase

Esterase activity was measured based on Zuo et al. (2010) with slight modifications. The reaction mixture contained 220 μl of 100 mM PBS buffer (pH 7.0), 25 μl of 5 mM of 4-nitrophenyl butyrate and 5 μl of the crude enzyme was incubated at 70°C for 10 min with shaking. Spectrophotometer reading at 410 nm was done immediately after incubation of the reaction mixture. One unit of esterase activity was defined as the rate at which 1 μmol of *p*-nitrophenol is liberated per min under assay conditions (Rúa et al., 1997).

### Statistical Analysis

The data were analyzed statistically using Microsoft Excel 2018. The means were compared using Two-way ANOVA to indicate any significant difference among parameters and the variables at *p* < 0.05. All experiments were done in triplicates unless otherwise mentioned.

## Results

### Isolation and Screening of Hydrocarbon-Degrading Strains

**FIGURE 1:**
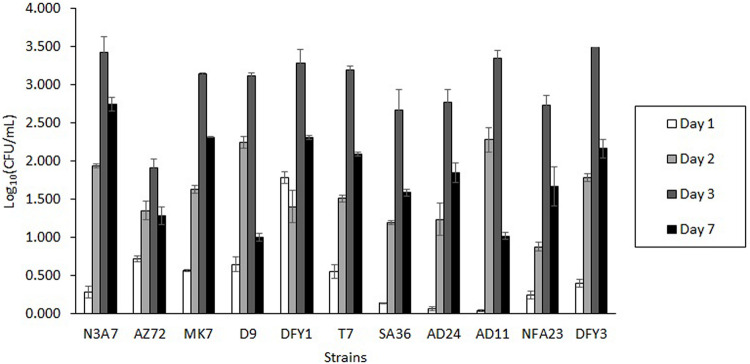
Growth profile of bacterial strains in the ocean water medium (OWM) supplemented with 2% (w/v) crude oil B (very hard). The bacteria were grown at 70°C and the colony forming unit (CFU) was determined.

Bacteria from various sampling sites were isolated based on their growth on Bushnell and Haas (BH) medium supplemented with different crude oils after 7 days incubation at 70°C. The ability of these bacteria to grow in the oil containing minimal BH medium suggested that these bacteria could utilize the crude oil as a carbon source for growth and degrade crude oil. Among them, AZ72, SA36, NFA23, AD11, AD24, N3A7, MK7, DFY1, T7, DFY3, and D9 showed different morphological characteristics and rapid growth in overnight culture were chosen for further test. During the screening, all strains were able to grow in the OWM containing very hard crude oil (crude oil B) (Figure 1) and showed maximum cell growth after 3 days of incubation and declined after 7 days. Degradation and dispersion of crude oil were observed in the bottles after 2 or 3 days in all cultures, in which the growth started to increase.

### Media Optimization

**FIGURE 2:**
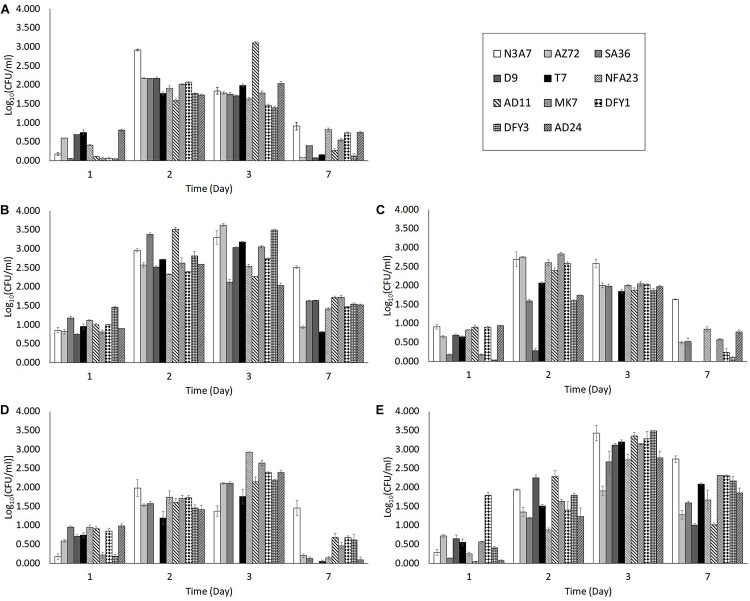
Bacterial growth in medium with **(A)** no organic nitrogen source (M1), **(B)** yeast extract (M2), **(C)** peptone (M3), **(D)** beef extract (M4), **(E)** with all three organic nitrogen sources (OWM). The growth of bacteria in the M2 medium was higher on average compared to M1, M3, and M4 medium. The growth in the M2 medium was also higher than the ocean water medium after 2 days. The degradation of crude oil was also supported in medium with yeast extract (*p* < 0.05).

Different organic nitrogen source was tested to optimize the condition of the growth medium. Among the organic nitrogen sources, yeast extract was the best for bacterial growth. The number of bacteria in yeast extract (M2 medium) was higher after 2 days incubation compared to the growth in peptone (M3 medium), beef extract (M4 medium), without organic nitrogen source (M1 medium), and with all three organic nitrogen sources (ocean water medium) on the same day (Figure 2). Strain AD11 showed the highest growth (3.512 CFU/ml) after 2 days of incubation in yeast extract. The number of bacteria in peptone (Figure 2C), beef extract (Figure 2D), and without organic nitrogen source (Figure 2A) was relatively lower than in yeast extract (Figure 2B). The growth in the OWM (Figure 2E) peaked only after 3 days of incubation. Moreover, peptone and beef extract showed a detrimental effect on strain D9, whereby the strain showed no growth after 2 days in beef extract and 3 days in peptone. However, some strains (N3A7 and AD11) showed high growth after 2 or 3 days incubation in M1 medium (Figure 2A). The bacterial growth in yeast extract, beef extract, and OWM was able to support crude oil degradation but not in peptone and medium without organic nitrogen source. Thus, due to the lower bacterial growth in beef extract and OWM at day 2, medium containing yeast extract was chosen as the best medium for bacterial growth and crude oil degradation experiment.

### Identification of the Bacteria

**FIGURE 3:**
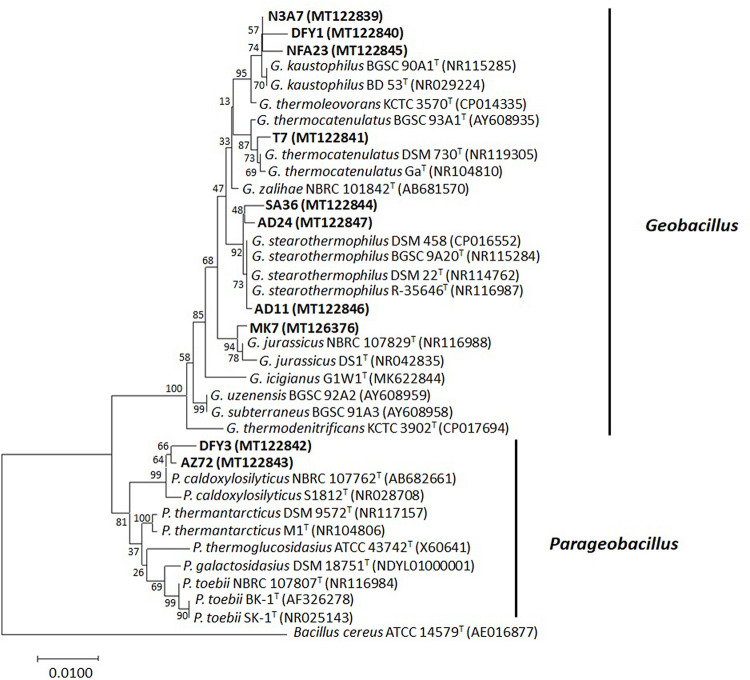
The 16S rRNA gene phylogenetic analysis of bacterial strains. The evolutionary history was inferred using the neighbor-joining method. The evolutionary distances were computed with *p-*distance method and 1,000 bootstrapped. The strains were aligned with closest relatives from GenBank. The accession numbers indicated within the bracket. The optimal tree with the sum of branch length = 0.18041597 was shown. The analysis involved 37 nucleotide sequences. There were a total of 1,204 positions in the final dataset. Bacterial strains used in this study are in bold. Sequence from *Bacillus cereus* ATCC 14579^T^ was used as an outgroup.

Approximately 1.5-kbp fragment of the 16S rRNA gene was amplified from the bacterial genomic DNA to identify the bacteria. The amplified fragments were compared to those sequences deposited in the GenBank database to determine the taxonomic identities. The 16S rRNA gene sequence of the bacteria displayed a similarity of 95–99% to the closest known species (Supplementary Table S1). Based on the 16S rRNA gene, a phylogenetic tree was constructed (Figure 3). Most of the bacteria belong to the phylum Firmicutes (*Geobacillus* spp. and *Parageobacillus* spp.). The eight bacterial strains belonging to the genus *Geobacillus* were closely related to *Geobacillus kaustophilus* (3), *Geobacillus stearothermophilus* (3), *Geobacillus thermocatenulatus* (1), and *Geobacillus jurassicus* (1). The two bacterial strains belonging to the genus *Parageobacillus* were both closely related to *Parageobacillus caldoxylosilyticus* (Figure 3).

Successively, based on the molecular identification and phylogenetic analysis, the 10 strains were identified as *Geobacillus kaustophilus* N3A7, DFY1, and NFA23, *Geobacillus jurassicus* MK7, *Geobacillus thermocatenulatus* T7, *Parageobacillus caldoxylosilyticus* DFY3 and AZ72, *Geobacillus stearothermophilus* SA36, AD11, and AD24. All sequences were submitted to the sequence database at the National Center for Biotechnology Information (NCBI).

### The Effect of Bacterial Growth and Rate of Degradation on the pH of the Medium

**FIGURE 4:**
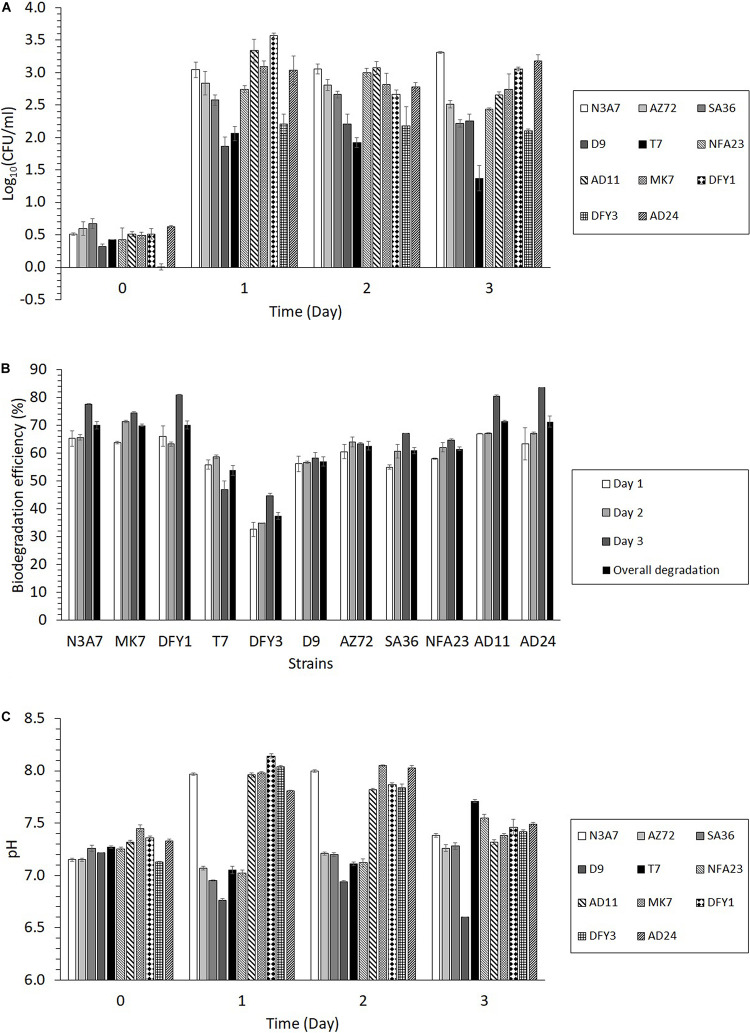
Growth, degradation efficiency, and changes in pH of growth medium during the degradation of crude oil. **(A)** Bacterial growth of the 11 strains in 2% (w/v) crude oil B for 3 days, **(B)** Crude oil biodegradation efficiency of the 11 strains from days 1 to 3 and overall degradation, **(C)** The pH of the medium during the degradation of crude oil (*p* < 0.05). The overall degradation is the average degradation from biodegradation efficiency on days 1 to 3. The crude oil biodegradation efficiency was calculated by comparing the peak areas of the inoculated sample with the peak areas of the control from GC–MS analysis.

The strains were grown in yeast extract (M2) medium containing 2% (w/v) crude oil B for 3 days. The growth and crude oil degradation of the strains were monitored by plate count and GCMS analysis, respectively. The initial growth of the 11 strains was not apparent; however, the growth of the strains increased after 1 day of incubation (Figure 4A). Some strains, such as N3A7 and D9, showed further induction in growth until day 3, while other strains declined after 2 days. Strains DFY1 and AD24 declined but slightly increased after 3 days, while the growth of strains SA36 and NFA23 increased after 2 days and then decreased.

Based on the results in Figure 4B, the biodegradation efficiency of the strains was between 33 and 67% on the first day. The biodegradation efficiency (35–71%) slightly increased on the second day, while the highest biodegradation efficiency was achieved after 3 days (45 to 84%). The highest overall biodegradation efficiency of crude oil among the 11 strains was exhibited by strains N3A7, MK7, DFY1, AD11, and AD24 (more than 70% oil degradation), the poorest degradation was observed by strain DFY3 (37%), while other strains showed moderate oil degradation (54 to 63%). The utilization of crude oil resulted in changes in the pH of the medium. The transformation of the oil shifted the pH toward slight alkalinity (pH 7.8–8.1) after 1 or 2 days of incubation with strains N3A7, MK7, DFY1, DFY3, AD24, and AD11 (Figure 4C). After 3 days of incubation, the pH decreased to pH 7.3–7.5. As for strains AZ72, SA36, NFA23, D9, and T7, the pH of the medium slightly reduced toward acidity. However, the pH of the medium of these strains continued to increase after 2 days between pH 7.3 to 7.7 except for strain D9, which further declined to pH 6.6 after 3 days.

### Preferential Degradation of Long-Chain *n*-Alkanes

The increase in the relative content of the carbon fractions ratio of ∑C_21–_/∑C_22+_ and (C_21_ + C_22_)/(C_28_ + C_29_) indicates that the strains prefer to utilize the long-chain hydrocarbon (Table 1). The ratio was mostly higher in strains N3A7, MK7, DFY1, AD11, and AD24 on different incubation periods. However, hydrocarbon ratio decreased slightly after 2 days of incubation of strains MK7 and AD11 and increased again on the third day. The ratio increased during incubation of strain DFY1, and N3A7 decreased after 2 days, while AD24 reduced after 3 days. The reduction demonstrated the utilization of the short-chain fraction after 2 or 3 days. The results also suggest that the strains would probably utilize the short-chain hydrocarbon when the contents of long-chain hydrocarbon were reduced. A minimal increase of the ∑C_21–_/∑C_22+_ and (C_21_ + C_22_)/(C_28_ + C_29_) ratio was observed in other strains during the incubation period. The minimum increase of ratios by strains T7, DFY3, D9, AZ72, SA36, and NFA23 was because of a lower rate of degradation compared to strains N3A7, MK7, DFY1, AD11, and AD24.

**TABLE 1:** The changes of *n*-alkane contents between hydrocarbon lower than C_22_ and higher than C_22_.

Strain	∑C_21–_/∑C_22+_			w(C_21_+C_22_)/w(C_28_ + C_29_)		
		
	Day 1	Day 2	Day 3	Day 1	Day 2	Day 3
Control	0.19	0.19	0.19	0.51	0.51	0.51
N3A7	0.41	0.36	0.23	0.62	0.59	0.58
MK7	0.37	0.27	0.43	0.71	0.40	0.77
DFY1	0.29	0.31	0.36	0.44	0.46	0.76
T7	0.30	0.29	0.32	0.37	0.38	0.39
DFY3	0.27	0.27	0.30	0.41	0.39	0.39
D9	0.31	0.36	0.30	0.42	0.47	0.42
AZ72	0.29	0.26	0.28	0.34	0.34	0.35
SA36	0.31	0.24	0.34	0.34	0.34	0.53
NFA23	0.27	0.29	0.36	0.36	0.39	0.47
AD11	0.29	0.28	0.41	0.39	0.36	1.02
AD24	0.27	0.38	0.23	0.40	0.65	0.59

**FIGURE 5:**
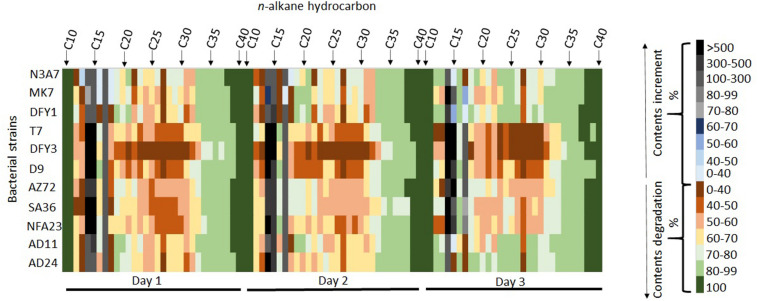
Heatmap showing degradation and increment of hydrocarbon contents during incubation. The percentage of hydrocarbon degradation and increment is represented by the different color scales on the right.

The heatmap shows the degradation of hydrocarbon by different strains with complete degradation of some long-chain hydrocarbon on different days of incubation (Figure 5). Interestingly, strains N3A7, MK7, DFY3, AZ72, SA36, NFA23, AD11, and AD24 could completely degrade C_38_ to C_40_, whereas strains DFY1 and T7 could completely degrade C_37_ to C_40_ by day 3 (Figure 5). Most of the strains could partially degrade the long-chain hydrocarbon above C_18_ and some short-chain hydrocarbon (C_12_–C_13_), especially on days 1 and 2. However, for a few strains such as N3A7, MK7, DFY1, AD11, and AD24, the biodegradation efficiency of most of the C_18_ and above hydrocarbon reached above 80% compared to the other strains by day 3. The majority of the strains showed low (1–68%) degradation of C_12_–C_13_ on days 1 and 2, but strains N3A7, DFY1, AD11, and AD24 were able to degrade more than 80% of C_12_ and C_13_ by day 3. All strains showed an increase in the content of C_14_, C_15_, and C_17_ on the first day of incubation. The composition of C_14_−C_17_ reduced during incubation. However, the composition was still higher than in control in most of the strains. Among the strains, N3A7 showed an increase of C_13_ (48%), C_16_ (9%), and C_18_ (11%), while MK7 showed an increase of C_18_ (8%) only on the first day (Figure 5). However, the trend of increment in the short-chain hydrocarbon content reduces throughout incubation in these strains. The rise of the short-chain fraction ensued from the degradation of the long-chain fraction and slower degradation of the short-chain fraction. The total ion chromatogram (TIC) of the crude oil degradation by each strain conducted in this study can be found in Supplementary Material (Supplementary Figures S1–S12).

### Degradative Enzyme Activities in the Biodegradation of Crude Oil

**FIGURE 6:**
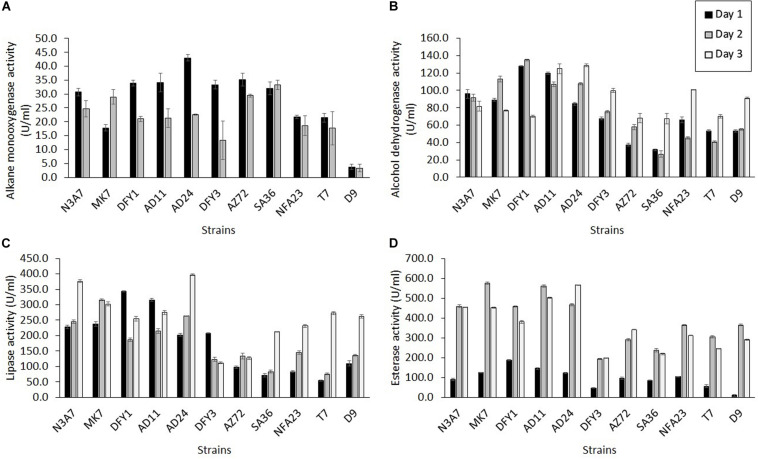
Quantification of enzyme activity. **(A)** Alkane monooxygenase, **(B)** alcohol dehydrogenase, **(C)** lipase, and **(D)** esterase activities during crude oil degradation by thermophilic bacterial strains (*p* < 0.05).

The alkane monooxygenase was induced in all strains during crude oil degradation, but the maximum induction was at different incubation periods (Figure 6A). All strains exhibited maximum alkane monooxygenase induction after 1 day incubation, while strains MK7 (29.0 U/ml) and SA36 (33.3 U/ml) showed maximum induction of this enzyme after 2 days incubation. The highest activity was exhibited by strain AD24 (43.0 U/ml) on the first day. Markedly high enzyme activity on the first day was also observed in other strains such as AZ72 (35.2 U/ml), AD11 (34.2 U/ml), DFY1 (33.9 U/ml), DFY3 (33.3 U/ml), SA36 (32.0 U/ml), and N3A7 (30.7 U/ml). The enzyme activity declined after 2 days of incubation, and by day 3 no activity was detected. The decline was attributed to a high removal of *n*-alkane between 74 and 84% (Figure 4B), especially by strains N3A7, MK7, DFY1, AD11, and AD24 on day 3.

All strains exhibited higher alcohol dehydrogenase activity than alkane monooxygenase (Figure 6B). Similarly, the maximum induction of alcohol dehydrogenase was on a different day of incubation. Strain N3A7 (96.1 U/mL) showed the maximum induction on the first day, and then the enzyme activity declined. The maximum induction of alcohol dehydrogenase was exhibited after 2 days by strains MK7 (113.0 U/mL) and DFY1 (134.9 U/mL) and then declined, while the maximum induction of enzyme by strains AD11 (124.9 U/mL), AD24 (128.7 U/mL), DFY3 (99.9 U/mL), AZ72 (68.3 U/mL), SA36 (67.4 U/mL), NFA23 (100.7 U/mL), T7 (70.1 U/mL), and D9 (91.2 U/mL) were after 3 days. The overall enzyme activity of strains N3A7, MK7, DFY1, AD11, and AD24 was the highest amongst the other strains. These strains also exhibited the highest average degradation in comparison to other strains despite some reduction in growth.

In comparison to the other two enzymes, esterase and lipase activity were significantly induced by all strains during crude oil degradation. The induction of esterase was initially slow but exhibited sudden increased after 2 days with maximum induction on the second day for the majority of the strains and then declined, except for strains AD24 (565.6 U/mL) and AZ72 (340.6 U/mL). Both of these strains showed maximum induction after 3 days. Similar to the other enzymes, strain N3A7, MK7, DFY1, AD11, and AD24 also showed a higher esterase activity on average compared to the other strains, especially after 2 or 3 days incubation. The results in Figure 6D indicated that the esterase activity of strains DFY3, AZ72, SA36, NFA23, T7, and D9 was significantly lower than the previously mentioned strains throughout the incubation period. The majority of the strains showed lower lipase production during the initial incubation, except for strains DFY1, AD11, and DFY3 (Figure 6C). The maximum lipase activity of strains DFY1, AD11, and DFY3 were induced to 343.5, 315.9, and 208.1 U/mL, respectively after 1 day incubation. Strain MK7 and AZ72 showed the highest lipase induction after 2 days, reaching up to 315.5 and 133.3 U/mL and then declined. The maximum lipase activity of strains N3A7, AD24, SA36, NFA23, T7, and D9 was induced after 3 days. An overall highest lipase activity was also observed in the five efficient degrader strains N3A7, MK7, DFY1, AD11, and AD24.

## Discussion

Bacteria can utilize various ranges of hydrocarbon (Hasanuzzaman et al., 2007; Binazadeh et al., 2009). The isolation of oil degraders, designated as N3A7, MK7, DFY1, T7, DFY3, D9, AZ72, SA36, NFA23, AD11, and AD24 on different types of crude oil with varying hardness shows that the bacteria can utilize a diverse range of hydrocarbon in the oil. The majority of the identified bacteria belonged to the genera *Geobacillus* and *Parageobacillus*. Strain D9 was identified previously as *Anoxybacillus geothermalis* D9 (MK615936.1) (Yusoff et al., 2020). The isolation and identification of these strains at 70°C suggest a possible role of these microorganisms to degrade crude oil in a high-temperature environment. The isolation of paraffin wax degrading microbes at 70°C was necessary because of the temperature of the extracted oil on the surface is approximately 70°C due to the high pour point (63°C) of crude oil B. Thus, making it vital to reduce the pour point just before the oil start to solidify at 63°C. Strains that could grow at high temperature (70°C) are suitable to be used in high-temperature oil reservoirs (Sood and Lal, 2008). Several members of *Geobacillus* sp. and *Anoxybacillus* sp. such as *Geobacillus caldoxylosilyticus* (Marchant et al., 2002), *Geobacillus kaustophilus* TERI NSM (Sood and Lal, 2008), *Geobacillus stearothermophilus* KTCC-B7S (Sorkhoh et al., 1993), and *Anoxybacillus rupiensis* (Xia et al., 2015) were found to utilize various alkane hydrocarbons. Some geobacilli has been transferred to *Parageobacillus* genus (e.g., *Parageobacillus caldoxylosilyticus*) (Aliyu et al., 2016).

The gradual increase of bacterial growth during incubation was associated with the adaptation of the bacteria toward the environment because crude oil is not an essential element for bacterial growth. During this period, enzymes are produced by the bacteria (Cappuccino and Welsh, 2017). The crude oil started to show signs of dispersion after 2 or 3 days of incubation, of which, the growth of all strains began to increase. The high growth obtained for all strains after 3 days of incubation reflects the capacity of these strains to use the various components in crude oil as substrates, allowing the degradation to occur when the bacteria were actively growing. Nutrients availability could also promote cell growth and degradation of crude oil by improving the production of the enzyme. The increase of bacterial growth and oil degradation with the addition of yeast extract (Okeke and Frankenberger, 2003; Mukred et al., 2008; Palanisamy et al., 2014; Farag et al., 2018), peptone (Mukred et al., 2008), and beef extract (Dixit and Pant, 2000) were previously reported. The addition of only yeast extract was shown to accelerate bacterial growth and allowed sufficient crude oil degradation as compared to other nutrients. As a source of vitamins and amino acids, yeast extract functioned as co-factor for the degradation of oil, which helped to improve enzyme production (El-Helow et al., 2000; Konsoula and Liakopoulou-Kyriakides, 2004). The absence of organic nitrogen sources, which resulted in insufficient nutrient levels in M1 medium resulted in low growth and crude oil degradation by the strains (Sihag et al., 2014). Some of the bacteria did show high growth in M1 medium due to the utilization of soluble short-chain alkanes, which are readily available than the larger molecules of long-chain alkanes (Sihag et al., 2014).

Bacterial growth and degradation of crude oil could also affect the pH of the growth medium. In the current results, the highest degradation was achieved by *Geobacillus kaustophilus* N3A7, *Geobacillus jurassicus* MK7, *Geobacillus kaustophilus* DFY1, *Geobacillus stearothermophilus* AD11, and *Geobacillus stearothermophilus* AD24 when the pH of the medium decreased toward neutral pH. The oxidation of *n*-alkane to alkanols reduces the pH from slightly alkaline during the initial 2 days incubation to neutral pH due to the accumulation of organic acids (Okpokwasili and James, 1995; Abdel-Megeed and Mueller, 2009; Darsa et al., 2014). Higher biodegradation efficiencies have been observed at a near-neutral pH (Abdel-Megeed and Mueller, 2009), as depicted in the present results. Mishra and Singh (2012) found the pH of the incubation medium was between 7.34 and 7.39, and after 10 days, the pH was slightly reduced to neutrality in the range of 7.0–7.3. Bacteria behaved differently in crude oil and, as a result, could produce alkaline by-products that shifted the pH toward alkalinity (Kim and Picardal, 2000; Qazi et al., 2013; Nwinyi et al., 2014; Meng et al., 2017). Thus, the increase in pH exhibited by some strains was due to their metabolites produced from crude oil.

The higher degradation of N3A7, MK7, DFY1, AD11, and AD24 stemmed from the higher biodegradation efficiency of the long-chain *n*-alkanes and the increase of short-chain alkanes, especially on days 1 and 2. The increase of the short-chain fraction in all strains (<C_21_) could probably be due to the conversion of the long-chain fraction into the short-chain fraction by bacterial enzymes (Hao and Lu, 2009; Sun et al., 2015). The bacteria and crude oil interaction is a complex biochemical process, which can affect the properties of crude oil directly or indirectly due to bacterial growth, enzymatic reactions, and metabolites (Hao and Lu, 2009). Bacteria and its metabolites can also alter the chemical properties of crude oil by decreasing the concentration of long-chain alkanes, which increased the short-chain fraction. The bacterial transformation of high molecular weight substrates through enzymatic reaction could cut the large aliphatic chains or internal linkage of the substrates into low molecular weight molecules, thus reducing the viscosity of the oil (Leon and Kumar, 2005). In the presently known biochemical mechanisms, alkanes are usually converted to alkanol, aldehyde, and then fatty acids, through enzymatic reactions (Ji et al., 2013). However, alkanes can also be produced by decarbonylation of aldehyde formed through the reduction of fatty acids. Based on the report by Park (2005), the reduction of hexadecenoic acid formed hexadecanal, which subsequently converted to pentadecane and carbon monoxide. Therefore, it could also be possible that the long-chain hydrocarbons were degraded into fatty acids, and some of the non-hydrocarbon products would undergo decarbonylation to form alkanes of shorter length. A higher light fraction is preferable as it implies that the viscosity, as well as production loss of the crude oil, is minimized. Thus, this indicates an added advantage of the 11 strains. Similar results were observed, whereby the degradation of long-chain alkanes increased the short-chain alkanes (Premuzic and Lin, 1999; Hao et al., 2004; She et al., 2011; Wenjie et al., 2012; Sun et al., 2015). Bioavailability, substrate conversion rate, and cell affinity toward hydrocarbon could also affect the biodegradation efficiency of the strains despite the decrease in bacterial growth. The biotransformation rate of hydrocarbon is not necessarily high if the ability to mass transfer the alkane substrate into the bacterial cell is the limiting factor (Boopathy et al., 1998). The bioavailability of hydrocarbon substrates could also affect the biodegradation efficiency of crude oil.

Contact between the hydrocarbon substrates and bacterial cells are essential before the molecular oxygen can be introduced by the functional oxygenases (Hua and Wang, 2014). The bioavailability of *n*-alkane C_18_ and above may be less compared to the other substrates owing to the slow mass transfer to the degrading microbes (Boopathy, 2000) and higher hydrophobicity (Ghazali et al., 2004). As a result, the effectiveness in mineralizing these hydrocarbon substrates was less, as the bacteria only partially degraded the substrates. Long-chain *n*-alkanes are less soluble (Sihag et al., 2014); therefore, they are less accessible for bacteria to metabolize compared to the short-chain. It is also possible that the alkane monooxygenase enzyme produced by the bacteria target other *n*-alkane hydrocarbons more than the C_18_ to C_36_ range. Apart from it, the rate of hydrocarbon transformation by bacterial cells was subjected to cell affinity (Rosenberg and Rosenberg, 1981). Cells with high attraction for hydrocarbon, degrade the hydrocarbon more effectively than those with low attraction. Hence, this could be the reason that strains N3A7, MK7, DFY1, AD11, and AD24 showed higher biodegradation efficiency of the hydrocarbon in crude oil despite the reduction in growth. Biosurfactant production could also be involved in the degradation of long-chain hydrocarbon by reducing the surface tension and making the hydrocarbon bioavailable for degradation by the bacteria. The reduction of the surface tension enhanced the solubilization of hydrocarbon in water or water in hydrocarbon (Patowary et al., 2016).

The degradative enzymes play a vital role in the degradation of hydrocarbon, and the respective genes encoding the enzymes had been identified in recent studies (Hassanshahian et al., 2012). The small and medium-chain alkanes are usually oxidized by methane monooxygenase and non-heme alkane monooxygenase, respectively (van Beilen et al., 1994). On the other hand, higher molecular weight hydrocarbons (>C_20_) are oxidized by many enzymes such as cytochrome P450s, alkane hydroxylase, flavin-binding monooxygenase, and LadA long-chain alkane monooxygenase (Nie et al., 2011; Singh et al., 2012). It is common for alkane degraders to degrade various alkane chain lengths due to the presence of multiple alkane hydroxylases with overlapping ranges of hydrocarbon. Hence, this allows the organism to utilize multiple substrate ranges and induce the enzyme at different times (van Beilen et al., 2002; Mishra and Singh, 2012; Jauhari et al., 2014). Some enzymes are secreted extracellularly, such as LadA from *Geobacillus thermodenitrificans* NG80-2. The long-chain alkane monooxygenase LadA is an extracellular protein capable of converting C_15_–C_36_ (Nie et al., 2011). Extracellular enzyme is more convenient to be used to lower pour point of crude oil as the cell do not need to be lysed to obtain the enzyme. The extracellular enzyme is more suitable for large scale production than intracellular enzyme as the enzyme is secreted outside the cell. The production of extracellular enzymes by the strains in this study caused the degradation of crude oil by altering the structure of alkane hydrocarbons to make it easier for bacteria to take up the metabolic intermediates across the cell membrane (Meng et al., 2017). Extracellular enzymes were also reported to accelerate the monooxygenation of diverse organic compounds selectively (Peter et al., 2011). Moreover, many organic pollutants are less soluble or toxic to bacteria. Thus, extracellular catalysis of the compounds is important. The extracellular enzyme activity that degrade long-chain hydrocarbon tested in the study is not specific. Genome and proteome analysis of the strains could further elucidate the specific genes encoding the enzymes for long-chain hydrocarbon degradation. Thus, the investigation on the specific genes responsible for the degradation of long-chain hydrocarbon through genome and proteome analysis, as well as biosurfactant production, should be carried out in the future.

The increase of alkane monooxygenase activity by the strains corresponds to the increase in alkane degradation efficiency until maximum enzyme activity was reached. Thus, indicating that alkane monooxygenase played an essential role in initiating of alkane degradation in crude oil. Surprisingly, the biodegradation efficiency of the strains continued to increase even after the enzyme activity had begun to decline. The increase in biodegradation efficiencies with declining enzyme activity might be associated with the activity of some intracellular enzymes involved in crude oil degradation (Olajuyigbe and Ehiosun, 2016). The break down of hydrocarbons into simpler molecules allow their assimilation and further degradation within the cell by intracellular enzymes, thus increasing degradation efficiency due to less hydrocarbon being present in the medium (Wasoh et al., 2019). A recent study by Yusoff et al. (2020) found that strain D9 exhibited extracellular alkane hydroxylase activity on the first day, but the activity reduced following the increase of the intracellular alkane hydroxylase activity. Further increase in biodegradation efficiency after the enzyme activity declined could also be due to the higher (more than 80%) removal of long-chain *n*-alkane (C_18_ to C_37_), especially by strains N3A7, MK7, DFY1, AD11, and AD24 on the 3^rd^ day of incubation. The reduction of residual *n*-alkane content resulted in a decline of extracellular alkane monooxygenase activity due to less substrate bioavailability. A similar observation was reported by Olajuyigbe and Ehiosun (2016) and Kadri et al. (2018a).

The biodegradation of crude oil is a continuous process. Therefore, there is also a possibility that degradation continues inside the cell as elucidated by Meng et al. (2017). Thus, reducing the extracellular alkane monooxygenase, alcohol dehydrogenase, lipase, and esterase activity due to the reduction of residual hydrocarbon present in the medium. As previously mentioned, the degradation of crude oil also depends on cell affinity to the hydrocarbon. Based on the results, even though strains AZ72, SA36, NFA23, DFY3, and T7 showed high or increased alkane monooxygenase, alcohol dehydrogenase, lipase, or esterase enzyme activities, but their biodegradation efficiency was lower compared to strains N3A7, MK7, DFY1, AD11, and AD24. Thus, this could be associated with the higher cell affinity of strains N3A7, MK7, DFY1, AD11, and AD24 toward the hydrocarbon leading to a more efficient uptake and degradation than the low-affinity cell. The higher alcohol dehydrogenase, lipase, and esterase activity after 2 or 3 days of incubation could be due to the increase of the degradation products such as alkanols, long-chain, and short-chain fatty acids which were produced through the oxidation of *n*-alkanes. The accumulation of these products resulted in the induction of the activity of these degradative enzymes. Previous studies also showed that less lipase enzyme was induced during the early incubation period, followed by a rapid increase in enzyme production when the hydrocarbon content decreased (Margesin et al., 2000; Elumalai et al., 2017).

In terms of cell growth, some strains showed a reduction in growth with increasing alcohol dehydrogenase, lipase, and esterase activity. Growth reduction could be due to the inhibitory effect of aliphatic alcohols and fatty acid on cell growth. Previous studies showed that alkanols could inhibit bacterial growth, despite being a very good alcohol dehydrogenase enzyme inducer (Alvarez et al., 2011). Thus, this could be the reason the degradative enzyme activity was higher despite low cell growth.

## Conclusion

This research presented evidence that suggests different nitrogen sources affect the capability of bacteria to degrade crude oil by means of enhancing or reducing the enzyme production. The increase of alkane monooxygenase activity corresponded to the increase in crude oil degradation efficiency until the maximum activity was reached. The evidence is consistent with the fact that alkane monooxygenase is used to initiate the degradation of *n*-alkane and that the degradation could also occur intracellularly. The ability of *Geobacillus kaustophilus* N3A7, *Geobacillus kaustophilus* DFY1, *Geobacillus jurassicus* MK7, *Geobacillus stearothermophilus* AD11, and *Geobacillus stearothermophilus* AD24 to efficiently degrade the crude oil (more than 70% biodegradation efficiency of crude oil) suggest that these strains produced the degradative enzymes, which effectively improve the degradation and uptake of hydrocarbon into bacterial cells for further biodegradation. The enzymatic degradation of long-chain *n*-alkane by cutting the large aliphatic chains or internal linkage of the substrates into short-chain *n-*alkane, and the possibility of short-chain *n*-alkane production through decarbonylation, reflect their potential application to reduce the pour point of crude oil and accelerate the biodegradation process.

## Data Availability Statement

The datasets generated for this study are available at the National Center for Biotechnology Information (NCBI) with the following accession number: MT122839, MT122842, MT126376, MT122841, MT122843, MT122844, MT122846, MT122845, MT122847, MT122840.

## Author Contributions

All authors listed have made a considerable, direct, and logical contribution to the work and approved it for publication. The experiments were designed by RR and MM. NA performed the experiments, analyzed the data and the GCMS results with the help from RR, MM, and MA. SS supervised the validation of bacterial identification. NA drafted the manuscript. RR, SS, and MA discussed the results, edit the manuscript, and approved the final version.

## Conflict of Interest

The authors declare that the research was conducted in the absence of any commercial or financial relationships that could be construed as a potential conflict of interest.

## Footnotes

1 https://www.ncbi.nlm.nih.gov/

## References

[B1] Abdel-Megeed A. Mueller R. (2009). Degradation of long-chain alkanes by a newly isolated *Pseudomonas frederiksbergensis* at low temperature. *Biorem. Biodiver. Bioavail.* 3 55–60.

[B2] Aliyu H. Lebre P. Blom J. Cowan D. De Maayer P. (2016). Phylogenomic re-assessment of the thermophilic genus *Geobacillus*. *Syst. Appl. Microbiol.* 39 527–523. 10.1016/j.syapm.2016.09.004 27726901

[B3] Alvarez L. Acevedo F. Illanes A. (2011). Induction of NAD+ dependent alcohol dehydrogenases with activity towards long-chain aliphatic alcohols in mesophilic, thermophilic and extreme thermophilic microorganisms. *Process Biochem.* 46 1342–1349. 10.1016/j.procbio.2011.03.002

[B4] Al-Yaari M. (2011). “Paraffin wax deposition: mitigation and removal techniques,” in *Proceedings of the SPE Saudi Arabia Section Young Professionals Technical Symposium*, Dhahran, 10.2118/155412-MS

[B5] Ameen F. Moslem M. Hadi S. Al-Sabri A. E. (2016). Biodegradation of diesel fuel hydrocarbons by mangrove fungi from Red Sea Coast of Saudi Arabia. *Saudi J. Biol. Sci.* 23 211–218. 10.1016/j.sjbs.2015.04.005 26981002 PMC4778521

[B6] Bai C. Zhang J. (2013). Thermal, macroscopic, and microscopic characteristics of wax deposits in field pipelines. *Energy Fuels* 27 752–759. 10.1021/ef3017877

[B7] Binazadeh M. Karimi I. A. Li Z. (2009). Fast biodegradation of long-chain n-alkanes and crude oil at high concentrations with *Rhodococcus* sp. Moj-3449. *Enzyme Microb. Technol.* 45 195–202. 10.1016/j.enzmictec.2009.06.001

[B8] Boopathy R. (2000). Factors limiting bioremediation technologies. *Bioresour. Technol.* 74 63–67. 10.1016/S0960-8524(99)00144-3

[B9] Boopathy R. Manning J. Kulpa C. F. (1998). A laboratory study of the bioremediation of 2,4,6-trinitrotoluene-contaminated soil using aerobic/anoxic soil slurry reactor. *Water Environ. Res.* 70 80–86. 10.2175/106143098X126919

[B10] Cappuccino J. G. Welsh C. T. (2017). *Microbiology: A Laboratory Manual*, 11th Edn London: Pearson Education.

[B11] Chahinian H. Sarda L. (2009). Distinction between esterases and lipases: comparative biochemical properties of sequence-related carboxylesterases. *Protein Peptide Lett.* 16 1149–1161. 10.2174/092986609789071333 19508178

[B12] Coon M. J. (2005). Omega oxygenases: nonheme-iron enzymes and P450 cytochromes. *Biochem. Biophys. Res. Commun.* 338 378–385. 10.1016/j.bbrc.2005.08.169 16165094

[B13] Darsa K. V. Thatheyus A. J. Ramya D. (2014). Biodegradation of petroleum compound using the bacterium *Bacillus subtilis*. *Sci. Int.* 2 20–25. 10.17311/sciintl.2014.20.25

[B14] Dixit V. S. Pant A. (2000). Hydrocarbon degradation and protease production by *Nocardiopsis* sp. NCIM 5124. *Lett. Appl. Microbiol.* 30 67–69. 10.1046/j.1472-765x.2000.00665.x 10728564

[B15] Doerffer J. W. (1992). *Oil Spill Response in the Marine Environment*, 1st Edn). Oxford: Pergamon Press.

[B16] El-Helow E. R. Abdel-Fattah Y. R. Ghanem K. M. Mohamad E. A. (2000). Application of the response surface methodology for optimizing the activity of an aprE-driven gene expression system in *Bacillus subtilis*. *Appl. Microbiol. Biotechnol.* 54 515–520. 10.1007/s002530000411 11092626

[B17] Elsharkawy A. M. Al-Sahhaf T. A. Fahim M. A. (2000). Wax deposition from Middle East crudes. *Fuel* 79 1047–1055. 10.1016/S0016-2361(99)00235-5

[B18] Elumalai P. Parthipan P. Karthikeyan O. P. Rajasekar A. (2017). Enzyme-mediated biodegradation of long-chain n-alkanes (C32 and C40) by thermophilic bacteria. *3 Biotech* 7:116. 10.1007/s13205-017-0773-y 28567628 PMC5451365

[B19] Fan Y. Wang J. Gao C. Zhang Y. Du W. (2020). A novel exopolysaccharide producing and long-chain n-alkane degrading bacterium *Bacillus licheniformis* strain DM-1 with potential application for in-situ enhanced oil recovery. *Sci. Rep.* 10:8519. 10.1038/s41598-020-65432-z 32444666 PMC7244480

[B20] Farag S. Soliman N. A. Abdel-Fattah Y. R. (2018). Statistical optimization of crude oil bio-degradation by a local marine bacterium isolate *Pseudomonas* sp. sp48. *J. Genet. Eng. Biotechnol.* 16 409–420. 10.1016/j.jgeb.2018.01.001 30733754 PMC6353655

[B21] Felsenstein J. (1985). Confidence limits on phylogenies: an approach using the bootstrap. *Evolution* 39 783–791. 10.1111/j.1558-5646.1985.tb00420.x 28561359

[B22] Ghazali F. M. Rahman R. N. Z. A. Salleh A. B. Basri M. (2004). Biodegradation of hydrocarbons in soil by microbial consortium. *Int. Biodeterior. Biodegrad.* 54 61–67. 10.1016/j.ibiod.2004.02.002

[B23] Gupta N. Rathi P. Gupta R. (2002). Simplified para-nitrophenyl palmitate assay for lipases and esterases. *Anal. Biochem.* 311 98–99. 10.1016/s0003-2697(02)00379-2 12441161

[B24] Hao R. Lu A. (2009). Biodegradation of heavy oils by halophilic bacterium. *Prog. Nat. Sci.* 19 997–1001. 10.1016/j.pnsc.2008.11.010

[B25] Hao R. Lu A. Zeng Y. (2004). Effect on crude oil by thermophilic bacterium. *J. Petrol. Sci. Eng.* 43 247–258. 10.1016/j.petrol.2004.02.017

[B26] Hasanuzzaman M. Ueno A. Ito H. Ito Y. Yamamoto Y. Yumoto I. (2007). Degradation of long-chain n-alkanes (C36 and C40) by *Pseudomonas aeruginosa* strain WatG. *Int. Biodeterior. Biodegrad.* 59 40–43. 10.1016/j.ibiod.2006.07.010

[B27] Hassanshahian M. Emtiazi G. Cappello S. (2012). Isolation and characterization of crude oil-degrading bacteria from the Persian Gulf and the Caspian Sea. *Mar. Pollut. Bull.* 64 7–12. 10.1016/j.marpolbul.2011.11.006 22130193

[B28] Hosseinipour A. Sabil K. M. Ekaputra A. A. Japper A. B. Ismail L. (2014). The impact of the composition of the crude oils on the wax crystallization. *Appl. Mech. Mater.* 625 196–200. 10.4028/www.scientific.net/AMM.625.196

[B29] Hua F. Wang H. Q. (2014). Uptake and trans-membrane transport of petroleum hydrocarbons by microorganisms. *Biotechnol. Biotechnol. Equip.* 28 165–175. 10.1080/13102818.2014.906136 26740752 PMC4684044

[B30] Jauhari N. Mishra S. Kumari B. Singh S. N. (2014). Bacteria-mediated aerobic degradation of hexacosane in vitro conditions. *Bioresour. Technol.* 170 62–68. 10.1016/j.biortech.2014.07.091 25125193

[B31] Jennings D. W. Breitigam J. (2010). Paraffin inhibitor formulations for different application environments: from heated injection in the desert to extreme cold arctic temperatures. *Energy Fuels* 24 2337–2349. 10.1021/ef900972u

[B32] Ji Y. Mao G. Wang Y. Bartlam M. (2013). Structural insights into diversity and n-alkane biodegradation mechanism of alkane hydroxylases. *Front. Microbiol.* 4:58. 10.3389/fmicb.2013.00058 23519435 PMC3604635

[B33] Kadri T. Magdouli S. Roussi T. Brar S. K. (2018a). Ex-situ biodegradation of petroleum hydrocarbons using *Alcanivorax borkumensis* enzymes. *Biochem. Eng. J.* 132 279–287. 10.1016/j.bej.2018.01.014

[B34] Kadri T. Rouissi T. Magdouli S. Brar S. K. Hegde K. Khiari Z. (2018b). Production and characterization of novel hydrocarbon degrading enzymes from *Alcanivorax borkumensis*. *Int. J. Biol. Macromol.* 112 230–240. 10.1016/j.ijbiomac.2018.01.177 29386098

[B35] Kaida N. (2012). *Biodegradation of Diesel by Local Isolate Bacillus pumilus Strain NHK.* Master’s Thesis, Universiti Putra Malaysia, Serdang, MY.

[B36] Kester D. R. Duedall I. W. Connors D. N. Pytkowicz R. M. (1967). Preparation of artificial seawater. *Limnol Oceanogr.* 12 176–179. 10.4319/lo.1967.12.1.0176

[B37] Kim S. Picardal F. W. (2000). A novel bacterium that utilizes monochlorobiphenyls and 4-chlorobenzoate as growth substrates. *FEMS Microbiol. Lett.* 185 225–229. 10.1111/j.1574-6968.2000.tb09066.x 10754252

[B38] Konsoula Z. Liakopoulou-Kyriakides M. (2004). Hydrolysis of starches by the action of α-amylase from *Bacillus subtilis*. *Process Biochem.* 39 1745–1749. 10.1016/j.procbio.2003.07.003

[B39] Kotani T. Yurimoto H. Kato N. Sakai Y. (2007). Novel acetone metabolism in a propane-utilizing bacterium. *Gordonia* sp. strain TY-5. *J. Bacteriol.* 189 886–893. 10.1128/JB.01054-06 17071761 PMC1797311

[B40] Kumar S. Stecher G. Tamura K. (2016). MEGA7: Molecular evolutionary genetics analysis version 7.0 for bigger datasets. *Mol. Biol. Evol.* 33 1870–1874. 10.1093/molbev/msw054 27004904 PMC8210823

[B41] Lee L. P. Karbul H. M. Citartan M. Gopinth S. C. B. Lakshmipriya T. Tang T. H. (2015). Lipase-secreting *Bacillus* species in an oil-contaminated habitat: promising strains to alleviate oil pollution. *Biomed Res. Int.* 2015:820575. 10.1155/2015/820575 26180812 PMC4477129

[B42] Leon V. Kumar M. (2005). Biological upgrading of heavy crude oil. *Biotechnol. Bioprocess Eng.* 10 471–481. 10.1007/BF02932281

[B43] Liu J. H. Jia Y. P. Chen Y. T. Xu R. D. (2013). Microbial treatment for prevention and removal of paraffin deposition on the walls of crude pipelines. *Indian J. Microbiol.* 53 482–484. 10.1007/s12088-013-0402-3 24426154 PMC3779288

[B44] Liu T. Wang F. Guo L. Li X. Yang X. Lin A. J. (2012). Biodegradation of n-hexadecane by bacterial strains B1 and B2 isolated from petroleum-contaminated soil. *Sci. China Chem.* 55 1968–1975. 10.1007/s11426-012-4618-6

[B45] Marchant R. Banat I. M. Rahman T. J. Berzano M. (2002). The frequency and characteristics of highly thermophilic bacteria in cool soil environments. *Environ. Microbiol.* 4 596–602. 10.1046/j.1462-2920.2002.00344.x 12366754

[B46] Margesin R. Zimmerbauer A. Schinner F. (2000). Monitoring of bioremediation by soil biological activities. *Chemosphere* 40 339–346. 10.1016/s0045-6535(99)00218-0 10665397

[B47] Meng L. Li H. Bao M. Sun P. (2017). Metabolic pathway for a new strain *Pseudomonas synxantha* LSH-7’: from chemotaxis to uptake of n-hexadecane. *Sci. Rep.* 7:39068. 10.1038/srep39068 28051099 PMC5209730

[B48] Michaud L. Giudice A. L. Saitta M. De Domenico M. Bruni V. (2004). The biodegradation efficiency on diesel oil by two psychrotrophic antarctic marine bacteria during a two-month-long experiment. *Mar. Pollut. Bull.* 49 405–409. 10.1016/j.marpolbul.2004.02.026 15325208

[B49] Mishra S. Singh S. N. (2012). Microbial degradation of n-hexadecane in mineral salt medium as mediated by degradative enzymes. *Bioresour. Technol.* 111 148–154. 10.1016/j.biortech.2012.02.049 22405754

[B50] Mukred A. M. Hamid A. A. Hamzah A. Wan Yusoff W. M. (2008). Enhancement of biodegradation of crude petroleum-oil in contaminated water by the addition of nitrogen sources. *Pak. J. Biol. Sci.* 11 2122–2127. 10.3923/pjbs.2008.2122.2127 19266926

[B51] Nei M. Kumar S. (2000). *Molecular Evolution and Phylogenetics.* New York, NY: Oxford University Press.

[B52] Nie Y. Liang J. Fang H. Tang Y. Q. Wu X. L. (2011). Two novel alkane hydroxylase-rubredoxin fusion genes isolated from Dietzia bacterium and the functions of fused rubredoxin domains in long-chain n-alkane degradation. *Appl. Environ. Microbiol.* 77 7279–7288. 10.1128/AEM.00203-11 21873474 PMC3194844

[B53] Nwinyi O. C. Kanu I. A. Tunde A. Ajanaku K. O. (2014). Characterization of diesel degrading bacterial species from contaminated tropical ecosystem. *Braz. Arch. Biol. Technol.* 57 789–756. 10.1590/S1516-8913201402250

[B54] Okeke B. C. Frankenberger W. T. Jr. (2003). Biodegradation of methyl tertiary butyl ether (MTBE) by a bacterial enrichment consortia and its monoculture isolates. *Microbial. Res.* 158 99–106. 10.1078/0944-5013-00181 12906382

[B55] Okpokwasili G. C. James W. A. (1995). Microbial contamination of kerosene, gasoline, and crude oil and their spoilage potentials. *Mater Org.* 29 147–156.

[B56] Olajuyigbe F. M. Ehiosun K. I. (2016). Assessment of crude oil degradation efficiency of newly isolated actinobacteria reveals untapped bioremediation potentials. *Bioremediat. J.* 20 133–143. 10.1080/10889868.2015.1113926

[B57] Palanisamy N. Ramya J. Kumar S. Vasanthi N. Chandran P. Khan S. (2014). Diesel biodegradation capacities of indigenous bacterial species isolated from diesel contaminated soil. *J. Environ. Health Sci. Eng.* 12:142. 10.1186/s40201-014-0142-2 25530870 PMC4271493

[B58] Park M. O. (2005). New pathway for long-chain n-alkane synthesis via 1-alcohol in *Vibrio furnissii* M1. *J. Bacteriol.* 187 1426–1429. 10.1128/JB.187.4.1426-1429.2005 15687207 PMC545631

[B59] Patowary K. Patowary R. Kalita M. C. Deka S. (2016). Development of an efficient bacterial consortium for the potential remediation of hydrocarbons from contaminated sites. *Front. Microbiol.* 7:1092. 10.3389/fmicb.2016.01092 27471499 PMC4943938

[B60] Peter S. Kinne M. Wang X. Ullrich R. Kayser G. Groves J. T. (2011). Selective hydroxylation of alkanes by an extracellular fungal peroxygenase. *FEBS J.* 278 3667–3675. 10.1111/j.1742-4658.2011.08285.x 21812933 PMC3586278

[B61] Premuzic E. T. Lin M. S. (1999). Induced biochemical conversions of heavy crude oils. *J. Petrol. Sci. Eng.* 22 171–180. 10.1016/S0920-4105(98)00066-7

[B62] Qazi M. A. Malik Z. A. Qureshi G. D. Hameed A. Ahmed S. (2013). Yeast extract as the most preferable substrate for optimized biosurfactant production by rhlB gene positive *Pseudomonas putida* SOL-10 isolate. *J. Bioremediat. Biodegrad.* 4:204 10.4172/2155-6199.1000204

[B63] Rosenberg M. Rosenberg E. (1981). Role of adherence in growth of Acinetobacter calcoaceticus RAG-1 on hexadecane. *J. Bacteriol.* 148 51–57. 10.1128/JB.148.1.51-57.1981 7287631 PMC216165

[B64] Rúa M. L. Schmidt-Dannert C. Wahl S. Sprauer A. Schmid R. D. (1997). Thermoalkalophilic lipase of *Bacillus thermocatenulatus* large-scale production, purification and properties: aggregation behavior and its effect on activity. *J. Biotechnol.* 56 89–102. 10.1016/S0168-1656(97)00079-5 9304872

[B65] Sadeghazad A. Ghaemi N. (2003). “Microbial prevention of wax precipitation in crude oil by biodegradation mechanism,” in *Proceedings of the SPE Asia Pacific Oil and Gas Conference and Exhibition*, Jakarta, 10.2118/80529-MS

[B66] Saitou N. Nei M. (1987). The neighbor-joining method: a new method for reconstructing phylogenetic trees. *Mol. Biol. Evol.* 4 406–425. 10.1093/oxfordjournals.molbev.a040454 3447015

[B67] Sakthipriya N. Doble M. Sangwai J. S. (2017). Enhanced microbial degradation of waxy crude oil: a review on current status and future perspective. *Int. J. Oil Gas Coal Technol.* 16 130–165. 10.1504/IJOGCT.2017.086315

[B68] Sharma N. Yasin F. F. Shahruddin S. Mohd Fadzil M. A. Rajantereh Y. Chandramalar A. V. M. (2016). Characterization of Malaysian waxy crude oils. *Petroleum Coal* 58 95–101.

[B69] She Y. H. Zhang F. Xia J. J. Kong S. Q. Wang Z. L. Shu F. C. (2011). Investigation of biosurfactant-producing indigenous microorganisms that enhance the residue oil recovery in an oil reservoir after polymer flooding. *Appl. Biochem. Biotechnol.* 163 223–234. 10.1007/s12010-010-9032-y 20652442

[B70] Sievers F. Higgins D. G. (2017). Clustal Omega for making accurate alignments of many protein sequences. *Protein Sci.* 27 135–145. 10.1002/pro.3290 28884485 PMC5734385

[B71] Sihag S. Pathak H. Jaroli D. P. (2014). Factors affecting the rate of biodegradation of polyaromatic hydrocarbons. *Int. J. Pure Appl. Biosci.* 2 185–202.

[B72] Singh S. N. Kumari B. Mishra S. (2012). “Microbial degradation of alkanes,” in *Microbial Degradation of Xenobiotics*, ed. Singh S. N. (Berlin: Springer), 439–469. 10.1007/978-3-642-23789-8_17

[B73] Sood N. Lal B. (2008). Isolation and characterization of a potential paraffin-wax degrading thermophilic bacterial strain *Geobacillus kaustophilus* TERI NSM for application in oil wells with paraffin deposition problems. *Chemosphere* 70 1445–1451. 10.1016/j.chemosphere.2007.08.071 17942139

[B74] Sorkhoh N. A. Ibrahim A. S. Ghannoum M. A. Radwan S. S. (1993). High-temperature hydrocarbon degradation by *Bacillus stearothermophilus* from oil-polluted Kuwaiti desert. *Appl. Microbiol. Biotechnol.* 39 123–126. 10.1007/BF00166860

[B75] Sun Y. Ning Z. Yang F. Li X. (2015). Characteristics of newly isolated *Geobacillus* sp. ZY-10 degrading hydrocarbons in crude oil. *Pol. J. Microbiol.* 64 253–263. 10.5604/01.3001.0009.2120 26638533

[B76] Tourova T. P. Sokolova D. S. Semenova E. M. Shumkova E. S. Korshunova A. V. Babich T. L. (2016). Detection of n-alkane biodegradation genes alkB and ladA in thermophilic hydrocarbon-oxidizing bacteria of the genera *Aeribacillus* and *Geobacillus*. *Microbiology* 85 693–707. 10.1134/S0026261716060199

[B77] Towler B. F. Jaripatke O. Mokhatab S. (2011). Experimental investigations of the mitigation of paraffin wax deposition in crude oil using chemical additives. *Petrol. Sci. Technol.* 29 468–483. 10.1080/10916460903394029

[B78] van Beilen J. B. Smits T. H. Whyte L. G. Schorcht S. Röthlisberger M. Plaggemeier T. (2002). Alkane hydroxylase homologues in gram positive strains. *Environ. Microbiol.* 4 676–682. 10.1046/j.1462-2920.2002.00355.x 12460275

[B79] van Beilen J. B. Wubbolts M. G. Witholt B. (1994). Genetics of alkane oxidation by *Pseudomonas oleovorans*. *Biodegradation* 5 161–174. 10.1007/bf00696457 7532480

[B80] Wang L. Tang Y. Wang S. Liu R. L. Liu M. Z. Zhang Y. (2006). Isolation and characterization of a novel thermophilic *Bacillus* strain degrading long-chain n-alkanes. *Extremophiles* 10 347–356. 10.1007/s00792-006-0505-504 16604274

[B81] Wasoh H. Veeraswamy K. Gunasekaran B. Shukor M. Y. (2019). Biodegradation of hydrocarbon sludge by *Pseudomonas* sp. strain UPM-KV. *J. Environ. Microbiol. Toxicol.* 7 10–15.

[B82] Watkinson R. J. Morgan P. (1990). Physiology of aliphatic hydrocarbon-degrading microorganisms. *Biodegradation* 1 79–92. 10.1007/BF00058828 1368149

[B83] Weisburg W. G. Barns S. M. Pelletier D. A. Lane D. J. (1991). 16S ribosomal DNA amplification for phylogenetic study. *J. Bacteriol.* 173 697–703. 10.1128/jb.173.2.697-703.1991 1987160 PMC207061

[B84] Wenjie X. Li Y. Ping W. Jianlong X. Hanping D. (2012). Characterization of a thermophilic and halotolerant *Geobacillus pallidus* H9 and its application in microbial enhanced oil recovery (MEOR). *Ann. Microbiol.* 62 1779–1789. 10.1007/s13213-012-0436-5

[B85] Xia W. Dong H. Zheng C. Cui Q. He P. Tang Y. (2015). Hydrocarbon degradation by newly isolated thermophilic *Anoxybacillus* sp. with bioemulsifier production and new alkB genes. *RSC Adv.* 5 102367–102377. 10.1039/c5ra17137g

[B86] Yan S. Wang Q. Qu L. Li C. (2013). Characterization of oil-degrading bacteria from oil-contaminated soil and activity of their enzymes. *Biotechnol. Biotechnol. Equip.* 27 3932–3938. 10.5504/BBEQ.2013.0050

[B87] Yong Y. C. Zhong J. J. (2010). Recent advances in biodegradation in China: new microorganisms and pathways, biodegradation engineering, and bioenergy from pollutant biodegradation. *Process Biochem.* 45 1937–1943. 10.1016/j.procbio.2010.04.009

[B88] Yusoff D. F. Raja Abd Rahman R. N. Z. Masomian M. Ali M. S. M. Leow T. C. (2020). Newly isolated alkane hydroxylase and lipase producing *Geobacillus* and *Anoxybacillus* species involved in crude oil degradation. *Catalysts* 10:851 10.3390/catal10080851

[B89] Zhang J. Zhang X. Liu J. Li R. Shen B. (2012). Isolation of a thermophilic, *Geobacillus* sp. SH-1, capable of degrading aliphatic hydrocarbons and naphthalene simultaneously, and identification of its naphthalene degrading pathway. *Bioresour. Technol.* 124 83–89. 10.1016/j.biortech.2012.08.044 22985850

[B90] Zhou J. F. Gao P. K. Dai X. H. Cui X. Y. Tian H. M. Xie J. J. (2018). Heavy hydrocarbon degradation of crude oil by a novel thermophilic *Geobacillus stearothermophilus* strain A-2. *Int. Biodeterior. Biodegrad.* 126 224–230. 10.1016/j.ibiod.2016.09.031

[B91] Zuo K. Zhang L. Yao H. Wang J. (2010). Isolation and functional expression of a novel lipase gene isolated directly from oil-contaminated soil. *Acta Biochim. Pol.* 57 305–311. 10.18388/abp.2010_2409 20931089

