# Supplementary material for: Microbial Biodegradation of Paraffin Wax in Malaysian Crude Oil Mediated by Degradative Enzymes
Source: Front Microbiol. 2020 Sep 8;11:565608. doi: 10.3389/fmicb.2020.565608 (PMC7506063; doi:10.3389/fmicb.2020.565608)
Supplement: Supplementary file 1 [file Data_Sheet_1.ZIP › Supplementary materials/Table S1.pdf]

Supplementary Material

Table S1 The comparison of 16S rRNA gene sequence of bacterial isolates from seawater and soil samples with the 16S rRNA gene sequence in GenBank.

	Sequence				
Isolates	GenBank accession no.[a]	Number of nucleotides[b]	Closest phylogenetic relative[c]	Score (gaps)[d]	Identity (%)[e]
N3A7	MT122839	1522	*Geobacillus kaustophilus* BGSC 90A1	2699 (1)	98.69
DFY3	MT122842	1524	*Parageobacillus caldoxylosilyticus* S1812	2691 (3)	98.87
MK7	MT126376	1527	*Geobacillus jurassicus* DS1	2730 (2)	99.40
T7	MT122841	1475	*Geobacillus thermocatenulatus* DSM 730	2533 (11)	98.14
AZ72	MT122843	1526	*Parageobacillus caldoxylosilyticus* S1812	2715 (0)	99.20
SA36	MT122844	1526	*Geobacillus stearothermophilus* BGSC 9A20	2584 (11)	97.56
AD11	MT122846	1515	*Geobacillus stearothermophilus* BGSC 9A20	2549 (17)	97.05
NFA23	MT122845	1533	*Geobacillus kaustophilus* BGSC 90A1	2447 (28)	95.70
AD24	MT122847	1516	*Geobacillus stearothermophilus* BGSC 9A20	2447 (41)	96.12
DFY1	MT122840	1520	*Geobacillus kaustophilus* BGSC 90A1	2601 (10)	97.76

[a] Accession number of each sequence was provided from GenBank database. [b] The number of 16S rRNA gene nucleotides from the combination of 8F and 1492R primers. [c] Closely related species from GenBank database. [d] The matching score with the closest phylogenetic relative has 0.0 E value and the number of gaps in bracket. [e] The percentage identity with the closest phylogenetic relative of bacteria.
