# Supplementary material for: Microbial Biodegradation of Paraffin Wax in Malaysian Crude Oil Mediated by Degradative Enzymes
Source: Front Microbiol. 2020 Sep 8;11:565608. doi: 10.3389/fmicb.2020.565608 (PMC7506063; doi:10.3389/fmicb.2020.565608)
Supplement: Supplementary file 1 [file Data_Sheet_1.ZIP › Supplementary materials/Figure S12.pdf]

Supplementary Material

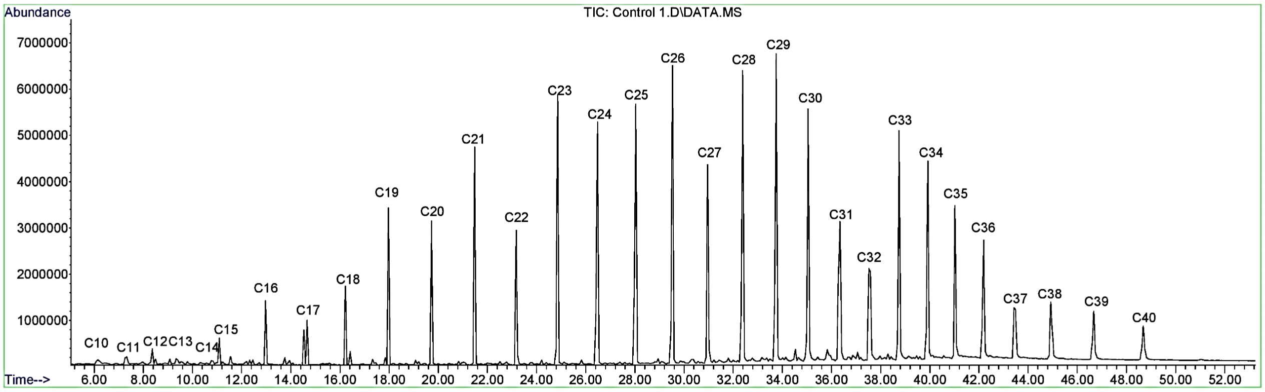

Figure S12A GCMS spectra of control without treatment on day 1.

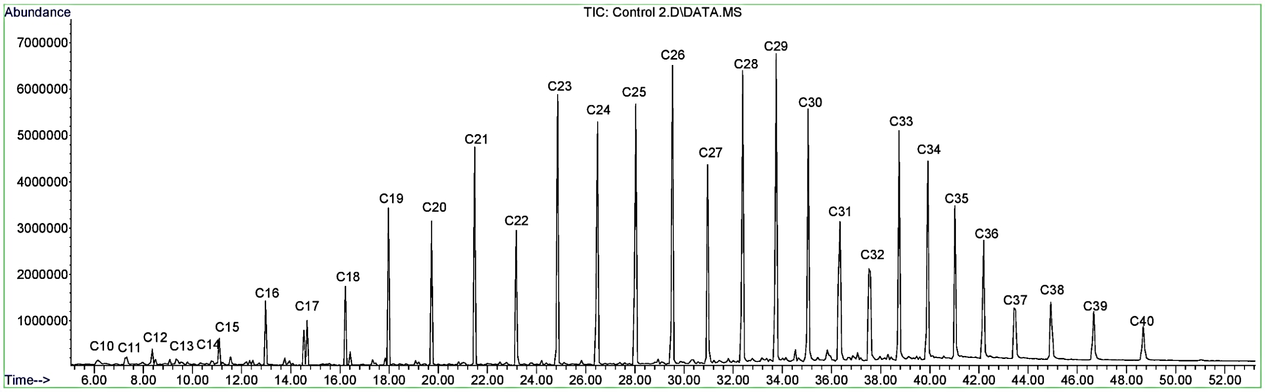

Figure S12B GCMS spectra of control without treatment on day 2.

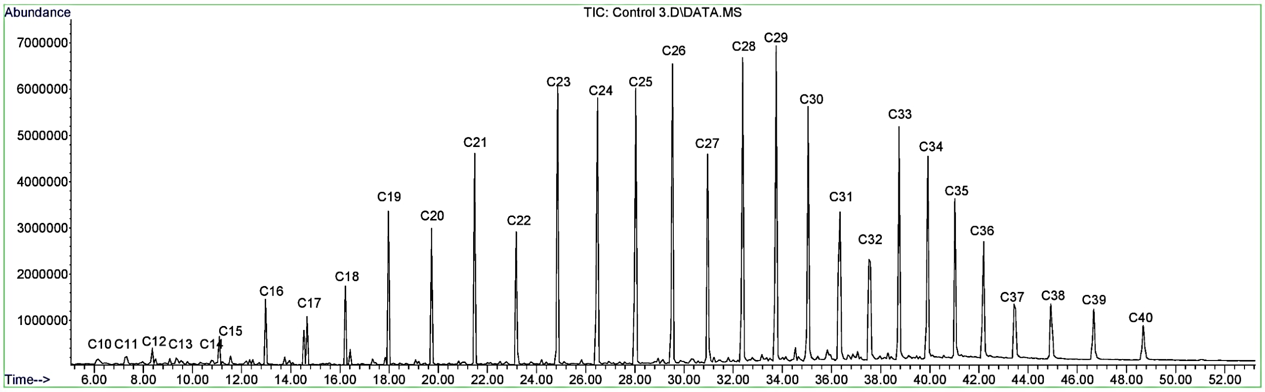

Figure S12C GCMS spectra of control without treatment on day 3.
