# Supplementary material for: Microbial Biodegradation of Paraffin Wax in Malaysian Crude Oil Mediated by Degradative Enzymes
Source: Front Microbiol. 2020 Sep 8;11:565608. doi: 10.3389/fmicb.2020.565608 (PMC7506063; doi:10.3389/fmicb.2020.565608)
Supplement: Supplementary file 1 [file Data_Sheet_1.ZIP › Supplementary materials/Figure S3.pdf]

Supplementary Material

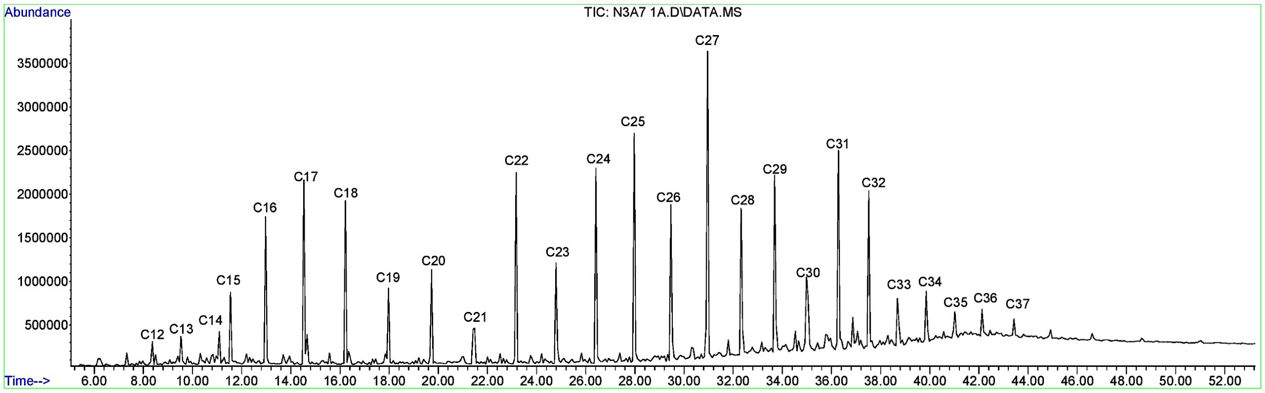

Figure S3A GCMS spectra of crude oil B treated with N3A7 at day 1.

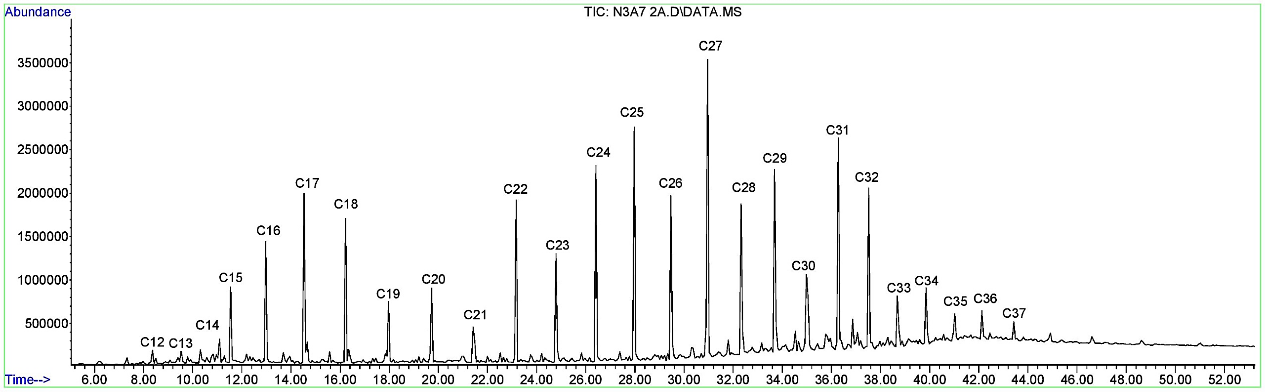

Figure S3B GCMS spectra of crude oil B treated with N3A7 at day 2.

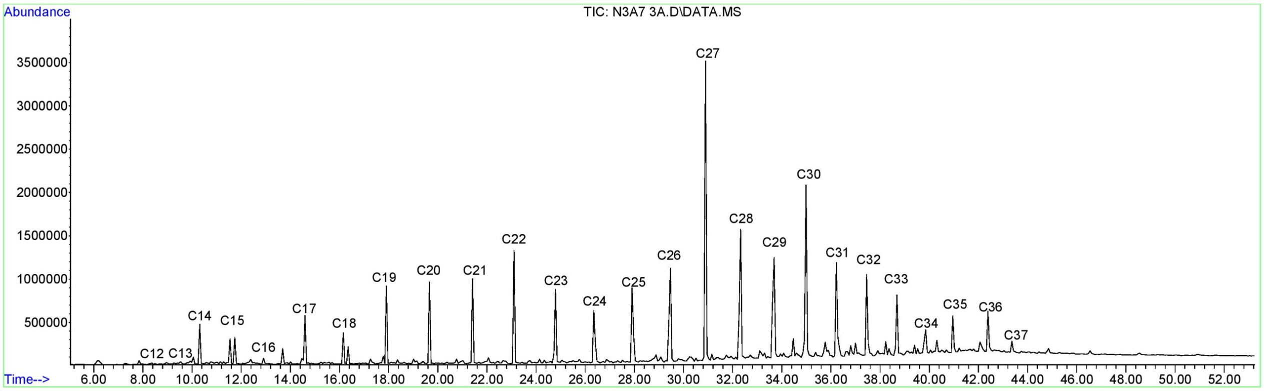

Figure S3C GCMS spectra of crude oil B treated with N3A7 at day 3.
